# Mechanisms and Potential Benefits of Neuroprotective Agents in Neurological Health

**DOI:** 10.3390/nu16244368

**Published:** 2024-12-18

**Authors:** Burcu Pekdemir, António Raposo, Ariana Saraiva, Maria João Lima, Zayed D. Alsharari, Mona N. BinMowyna, Sercan Karav

**Affiliations:** 1 Department of Molecular Biology and Genetics, Çanakkale Onsekiz Mart University, Çanakkale 17100, Turkey; burcupekdemir0@gmail.com; 2 CBIOS (Research Center for Biosciences and Health Technologies), Universidade Lusófona de Humanidades e Tecnologias, Campo Grande 376, 1749-024 Lisboa, Portugal; 3 Research in Veterinary Medicine (I-MVET), Faculty of Veterinary Medicine, Lisbon University Centre, Lusófona University, Campo Grande 376, 1749-024 Lisboa, Portugal; ariana.saraiva@ulusofona.pt; 4 CERNAS Research Centre, Polytechnic University of Viseu, 3504-510 Viseu, Portugal; mjoaolima@esav.ipv.pt; 5 Department of Clinical Nutrition, Faculty of Applied Medical Sciences, University of Tabuk, P.O. Box 741, Tabuk 71491, Saudi Arabia; zalsharari@ut.edu.sa; 6 College of Education, Shaqra University, Shaqra 11911, Saudi Arabia; m.mwena@su.edu.sa

**Keywords:** anti-inflammatory, antioxidant, neurodegenerative, neuroplasticity, neuroprotection

## Abstract

The brain contains many interconnected and complex cellular and molecular mechanisms. Injury to the brain causes permanent dysfunctions in these mechanisms. So, it continues to be an area where surgical intervention cannot be performed except for the removal of tumors and the repair of some aneurysms. Some agents that can cross the blood–brain barrier and reach neurons show neuroprotective effects in the brain due to their anti-apoptotic, anti-inflammatory and antioxidant properties. In particular, some agents act by reducing or modulating the accumulation of protein aggregates in neurodegenerative diseases (Alzheimer’s disease, Parkinson’s disease, Huntington’s disease, Amyotrophic lateral sclerosis, and prion disease) caused by protein accumulation. Substrate accumulation causes increased oxidative stress and stimulates the brain’s immune cells, microglia, and astrocytes, to secrete proinflammatory cytokines. Long-term or chronic neuroinflammatory response triggers apoptosis. Brain damage is observed with neuronal apoptosis and brain functions are impaired. This situation negatively affects processes such as motor movements, memory, perception, and learning. Neuroprotective agents prevent apoptosis by modulating molecules that play a role in apoptosis. In addition, they can improve impaired brain functions by supporting neuroplasticity and neurogenesis. Due to the important roles that these agents play in central nervous system damage or neurodegenerative diseases, it is important to elucidate many mechanisms. This review provides an overview of the mechanisms of flavonoids, which constitute a large part of the agents with neuroprotective effects, as well as vitamins, neurotransmitters, hormones, amino acids, and their derivatives. It is thought that understanding these mechanisms will enable the development of new therapeutic agents and different treatment strategies.

## 1. Introduction

**Figure 1:**
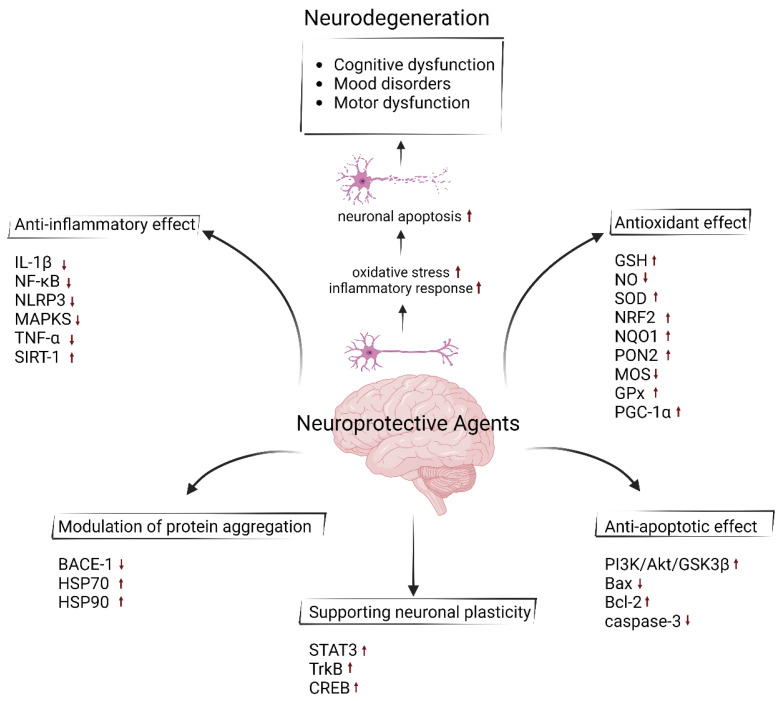
Neuroprotective effect of agents with their properties of anti-inflammatory, antioxidant, anti-apoptotic, modulating neuroplasticity and preventing the protein aggregation using different mechanisms of action. Upward arrows represent an increase in the activity or synthesis of the indicated molecules, whereas downward arrows indicate a decrease. (Created by Biorender at 14 November 2024; https://www.biorender.com).

The brain remains an area where surgical intervention cannot be performed, except for the removal of tumors and repair of some aneurysms. For this reason, brain injuries such as concussions and strokes are thought to be almost impossible to reverse [1]. In neuroscience, it is necessary to understand the cellular and molecular events in chronic events causing neuronal death and serious neurocognitive impairment following hypoxia, ischemia, hypoglycemia, and epileptogenic crises [2]. However, it is very important to understand the function of agents that provide neuroprotection in these inaccessible regions of the brain (Figure 1). Most agents can cross the blood–brain barrier (BBB) and reach individual neurons. Neuroprotective agents are used to protect neuronal structure and function and reverse or prevent some of the damage by reducing or stopping neuron loss [3].

It is known that cellular homeostasis decreases with cellular aging. The nervous system is sensitive to oxidative damage and stress due to unsaturated fats and iron. Decreasing homeostasis causes reactive oxygen species (ROS) production and accumulated free radicals affect negatively mitochondrial and synaptic function [4]. Following this process, impaired cell functions cause neuroinflammation and oxidation. Neuroinflammation involves inflammatory responses of glial cells such as microglia or astrocytes. These responses, which are normally aimed at protecting the brain, can lead to neuronal damage in chronic cases or in cases of excessive neuroinflammation. With inflammation, the release of proinflammatory cytokines increases and neuronal apoptosis is triggered [5].

In some neurodegenerative diseases such as Alzheimer’s disease (AD), Parkinson’s disease (PD), Huntington’s disease (HD), and Amyotrophic Lateral Sclerosis (ALS), irreversible damage to neurons is observed after the accumulation of toxic protein aggregates. Therefore, these diseases are characterized as chronic and progressive disorders with neuronal loss in motor, sensory, and cognitive systems. The positive effect of bioactive components with neuroprotective effects in cognitive deficit processes, such as dementia due to neuronal loss, is quite remarkable. Some neuroprotective agents can help prevent the accumulation of toxic protein aggregates [6]. At the same time, neuroprotective agents that help protect the nervous system from damage, have positive effects on improving mitochondrial function and scavenging free radicals, as well as inhibiting the inflammatory process and thus reducing neuronal death [7]. At the same time, although the primary function of these agents is to protect neurons, some neuroprotective agents can also promote cell proliferation by strengthening repair mechanisms in the brain. Because neuroprotective agents target improvements in various neurological conditions, they also provide a suitable environment for the re-establishment of the structural and functional organization of neurovascular networks. This ability, known as neuroplasticity, is a complex physiological process in which the brain continually reorganizes itself by establishing new neural connections throughout life [8]. This process is known as a mechanism for an organism to accomplish ongoing changes in environmental and mental conditions in addition to the genetic information accumulated during evolution for its survival [9]. Human growth and aging alter sense, memory, and behavior in the brain. Apart from these factors, disorders and injuries of the central nervous system (CNS) also change cognition. Neurogenesis, synaptogenesis, or neurochemical changes are included in these changes in the CNS. Unlike plasticity in a healthy brain, compensatory plasticity in a damaged brain begins in critical situations related to inflammation, edema, apoptosis, degeneration of nerve fibers, and metabolic disorders [8]. Neurogenesis, which refers to the production process of new neurons, usually occurs intensively during early brain development, such as the embryonic and fetal periods [10]. In adults, the formation of new neurons occurs in certain areas of the brain, such as the hippocampus. It contributes to learning and memory processes. It also regulates emotional responses and facilitates the management of conditions such as stress and depression. In the process of neurogenesis, progenitor cells are formed by the division of neural stem cells. New mature neurons are formed with the differentiation of progenitor cells. The new mature neurons then migrate to appropriate locations in the brain and are integrated into existing neuronal circuits in their functional synapses, thus integrating new neurons. These processes increase the flexibility of the brain, contributing to the development of new skills and the adaptation of the brain [11]. Synaptogenesis, known as the formation of new synapses between neurons and the strengthening of existing synapses, is another process that contributes to neuroplasticity and continues in every part of the brain from birth throughout life. In this process, it provides learning and memory processes by allowing neurons to communicate with each other.

Along with pharmacological drugs such as memantine and riluzole, known as neuroprotective agents, bioactive compounds found in nature can also have neuroprotective effects by playing important roles in the regenerative processes and plasticity of brain tissue [12]. Examples of natural compounds are polyphenols, vitamins, neurotransmitters, amino acids, and their derivatives, and omega-3 fatty acids (Table 1). Polyphenols are the most abundant antioxidants in the diet and are 10 times and 100 times higher than the intake of vitamin C and vitamin E, respectively, which are known for their antioxidant properties. It is known that flavonoids, which constitute a large part of polyphenols, support synaptogenesis and neurogenesis by inhibiting oxidative stress and neuroinflammation [13]. These bioactive compounds, which have a wide range of biological properties and neuroprotective effects such as antioxidant, anti-inflammatory, antimicrobial, and anticancer activities, are found in plants, foods, and other natural sources. However, many of them have not yet been fully elucidated. In particular, more clinical studies are needed on the protection provided against neurodegenerative diseases by polyphenols with strong antioxidant properties such as chlorogenic acid, ferulic acid, and caffeic acid in coffee, one of the most consumed beverages in the world. In addition, the relationship and synergistic effect of these compounds are also important. Although coffee is not a significant source of ferulic acid, ferulic acid is formed during the metabolism of chlorogenic acid. Therefore, coffee consumption can indirectly increase ferulic acid intake. Caffeic acid is a powerful neuroprotective agent that is effective in preventing oxidative stress and inflammation. Although it is known that caffeic acid can be taken into the body regularly through the diet, research on the use and reliability of supplements for higher doses needs to be expanded. Due to their contribution to neuroprotection and cognitive health, the inclusion of these bioactive compounds in the diet through supplements has recently increased the demand for new products [14]. Especially as the global population ages, functional foods, nutraceuticals, and pharmaceuticals rich in bioactive compounds with neuroprotective effects have become quite popular in the food and pharmaceutical industries to prevent age-related diseases [15].

**Figure 2:**
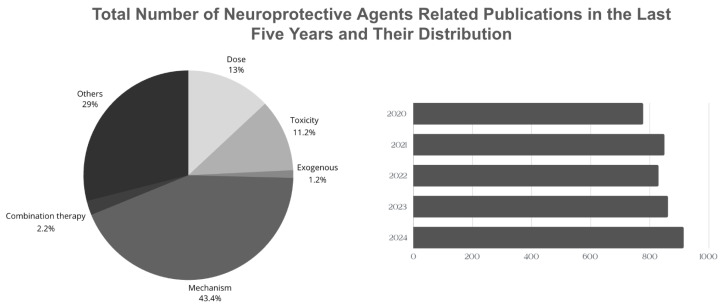
Comparison of the total number of neuroprotective agents-related publications in the last five years.

According to the Web of Science (WoS) database, approximately 4247 articles related to the “neuroprotective agents” topic are published annually (Figure 2) [139]. Although there are significant studies to explain the effects and biological mechanisms of these agents on health, studies based on the evaluation of the necessary consumption of these agents taken with the diet or used as supplements are relatively low. In addition, the development of therapeutic agents that target a single step in more than one molecular mechanism that plays a role in brain damage may not always be an effective treatment method. Therefore, the focus is on more than one therapeutic agent that can target synergistic pathways. However, there are difficulties such as identifying such agents and determining their toxicity. Therefore, studies on the potential interaction between combined nanoparticle designs, functional nutrients, and neuroprotective agents and the long-term effects of these agents on the organism after they cross the blood–brain barrier and reach the target area are of great interest.

This review includes current studies on the neuroprotective mechanisms of various agents documented in the literature from the classes of polyphenols, vitamins and vitamin-like compounds, hormones, neurotransmitters, amino acids and their derivatives, and proteins, and their exogenous sources of intake. It also provides an overview of geographical dietary habits and the daily requirements of these agents. A pie chart showing the distribution of articles containing the keywords ‘exogenous’, ‘non-exogenous’, ‘combination’ ‘toxicity’, ‘daily intake’, and ‘mechanism’ is shared (Figure 2). Therefore, current reviews focusing on the complex and interacting mechanisms of neuroprotective agents are extremely important in terms of providing insight into the daily dose, toxicity, or the results of combined supplementation of these agents. Based on this, instead of focusing only on agents obtained from natural sources, the literature should also be expanded on sources that cannot be obtained from natural sources but whose synthesis in the body can be increased by other natural source agents.

## 2. Polyphenols

### 2.1. Curcumin

Curcumin (diferuloylmethane) is the major active ingredient of turmeric (*Curcuma longa*), a dietary spice used in Indian cuisine and medicine [16]. Curcumin was first chemically characterized in 1910. It constitutes 2–8% of the turmeric preparation and has a low molecular weight. It exhibits various anti-inflammatory and anticancer properties after oral or topical administration [17]. The pro-inflammatory cytokine interleukin-1 beta (IL-1β) is known to activate nuclear factor-kappa B (NF-κB). This in turn activates proteins involved in matrix degradation, inflammation, and apoptosis. A study has identified curcumin as a phytochemical that inhibits NF-κB activation caused by IL-1β [21]. In myeloma cells, curcumin has also been shown to inhibit a signal transducer and activator of transcription 3 phosphorylation, thereby suppressing interleukin-6 (IL-6) production [19]. It has also been shown to have immunomodulatory effects, including activation of host macrophages and natural killer cells [18]. At the same time, curcumin, which is in the polyphenol group, has a strong antioxidant capacity at neutral and acidic pH [17]. It reverses the inhibition of DNA repair enzymes after oxidative DNA damage and relieves mitochondrial dysfunction caused by oxidative stress. It increases the antioxidant capacity of the brain by increasing glutathione levels and the activity of antioxidant enzymes such as superoxide dismutase and catalase [16]. Nitric oxide (NO) is a short-lived, lipid-soluble molecule produced from L-arginine by various NADPH (Nicotinamide Adenine Dinucleotide Phosphate)-dependent enzymes known as NO synthases (NOS). NO plays a physiological role in immune defense and intracellular signaling, as well as biological roles such as vasorelaxation and neurotransmission. However, NO is a free radical species because it has an unpaired electron. Although its bioactivity is associated with the production of many reactive intermediates, most of these reactive nitrogen species can damage DNA and prevent DNA repair. Studies have shown that the inducibility of macrophage NOS activity is inhibited by curcumin [17].

Curcumin is an important neuroprotective agent because of its antioxidant properties, protecting substantia nigra neurons from reactive oxygen species, improving striatal dopamine levels, and chelating Fe^2+^ in PD models. Curcumin derivatives with improved absorption have shown enhanced neuroprotective effects, although they have low bioavailability. These findings suggest that curcumin and its analogs could be promising therapeutic agents for reducing oxidative and nitrosative stress in neurodegenerative disorders such as PD [16]. Considering that curcumin can also cross the BBB, most studies investigating its therapeutic effects in neurodegenerative diseases focus on PD. According to epidemiological studies, the lower prevalence of AD/PD in India has been associated with higher turmeric/curcumin consumption among Asian Indians compared to Caucasians [140]. Curcumin has shown neuroprotective effects on both the brain and peripheral nerves in numerous in vivo studies. Due to these properties, it is considered to have significant potential for treating spinal cord injuries (SCIs). SCIs have two phases: The primary phase involves mechanical and structural damage and is irreversible, and the secondary phase consists of a series of systemic and local neurochemical and physiological changes. These changes are accompanied by edema, ischemia, cytokine production, inflammation, free radical damage, glial scarring, apoptosis, and necrosis. Secondary injury worsens over time and needs therapeutic intervention. Curcumin can be used as a therapeutic agent for SCI due to its ability to reduce secondary injuries [20].

### 2.2. Resveratrol

Resveratrol as a natural bioactive compound is found in grape skins and produced by some spermatophytes, such as grapevines, in response to injury. It is known as phytoalexin which has the ability to modulate lipid metabolism, prevent platelet aggregation, and inhibit the oxidation of low lipoproteins [24]. Additionally, resveratrol has antioxidant, anti-apoptotic, and anticancer characteristics and has positive effects on cardiovascular diseases, neurodegenerative diseases, diabetes, and infectious diseases [25].

Sirtuin 1 (SIRT1) activation modulates mitochondrial biogenesis, cellular stress responses, and antioxidant defenses. Nuclear factor erythroid 2-related factor 2 (NRF2) activation increases the expression of antioxidant enzymes such as oxygenase-1 and NADPH quinone oxidoreductase 1. Resveratrol has a neuroprotective role in ischemic stroke (IS), a complex and lethal condition involving multiple steps including BBB disruption, excitotoxicity, oxidative stress, neuroinflammation, and microglia overactivation, by activating NRF2 and SIRT1 pathways. Resveratrol enhances cellular survival during IS and reduces inflammation and apoptosis by neutralizing ROS [22].

Resveratrol has also been promising in the treatment of neurodegenerative diseases including Multiple sclerosis (MS). MS is characterized by myelin loss. The therapeutic effect of resveratrol for this disease, which causes significant neurological problems in young adults, was investigated in a study using the cuprizone model of MS. Oral resveratrol administration to cuprizone-poisoned C57Bl/6 mice demonstrated pro-remyelination effects despite the low bioavailability of resveratrol. Resveratrol significantly improved mitochondrial dysfunction by reducing oxidative stress. It indicated anti-inflammatory and anti-apoptotic effects by down-regulating NF-κB signaling and was shown to improve balance and motor coordination by promoting remyelination [23] and stimulating neurogenesis. It also protects neurons in AD by protecting against the harmful effects of oxidative stress through SIRT1 overexpression and suppressing the apoptotic effects of p53 [28]. In experimental autoimmune encephalomyelitis mice with optic neuritis, it was demonstrated that retinal ganglion cell loss was considerably reduced after intranasal consumption of resveratrol, which could be used as a potential neuroprotective treatment for optic neuritis [27].

### 2.3. Green Tea Polyphenols

Green tea is obtained by collecting the leaves of the *Camellia sinensis* plant and roasting and drying them quickly [32]. Green tea, which is widely consumed in Japan, China, and other Asian countries, is richer in polyphenols than black tea and also contains essential natural and bioactive compounds such as caffeine and amino acids. Known for its antioxidant and anti-inflammatory properties, green tea has also been demonstrated to have anticancer activity by playing a role in mechanisms including suppressing NF-κB, inflammasome, and IL-1β secretion [31,32]. Green tea polyphenols include catechins, which make up 30% of the dry weight of tea leaves. Examples of catechin types are epicatechin (EC), epicatechin gallate (ECG), and epigallocatechin (EGC). However, the most abundant catechin type that has been studied is epigallocatechin gallate (EGCG) [32]. After oral administration, its metabolites are excreted in the urine and EGCG is mainly absorbed in the intestine. It passes through the BBB and reaches the CNS even at very low concentrations. Therefore, many studies on EGCG have also been on its neuroprotective effects. As a result of these studies, it has been shown that it can provide neuroprotection in brain and spinal cord injuries, reduce Aβ plaque formation and tau protein phosphorylation seen in AD pathogenesis, and improve cognitive functions. It is also known that catechins have anti-apoptotic properties by regulating mitochondrial membrane permeability and suppressing the expression of pro-apoptotic genes [29].

### 2.4. Quercetin

Quercetin, which has great importance among polyphenols, is known as one of the most active plant-based antioxidants. Quercetin can be obtained through a diet of foods such as apples, buckthorn, berries, potatoes, and grapes [34,141].

There are views that quercetin is mutagenic and carcinogenic. This controversial issue has been recently concluded with in vitro and in vivo studies that quercetin is antimutagenic and not carcinogenic [141]. In chemical safety tests, the results of in vitro genotoxicity tests do not always mean that it has carcinogenic effects in vivo. The genotoxicity results that were positive for quercetin were negative in the in vivo carcinogenicity results. This is thought to be due to the limited absorption and bioavailability of quercetin after it is metabolized in the digestive system [33]. Especially in recent studies, it has been shown that quercetin, like bioactive compound sources including curcumin and resveratrol, also exhibits chemopreventive properties. It is thought that quercetin, which regulates many molecular mechanisms, has therapeutic effects for anticancer treatments [26]. In cancer treatments where dietary supplements are considered important in addition to chemotherapy, quercetin is seen as an anticancer agent due to its strong antioxidant and anti-inflammatory properties, low toxicity, and side effects [33].

In addition to these therapeutic effects, the neuroprotective effect of quercetin has also been examined in many studies. Quercetin has been considered to increase neurogenesis by regulating many pathways in the CNS [34]. Tumor Necrosis Factor-alpha (TNF-α), which is responsible for cell signaling in apoptosis and necrosis processes after acute inflammation, has been inhibited by quercetin and has an anti-inflammatory effect. It has been shown that the expression of the protein Paraoxonase 2 (PON2), which is known as an antioxidant and protects mitochondria from oxidative damage due to its high abundance in mitochondria, is increased by quercetin. In a study on AD treatment, it was shown that hyperphosphorylation, which disrupts the function of the tau protein, is regulated by quercetin using mitogen-activated protein kinases and PI3K/Akt/GSK3β signaling pathways. It was indicated that apoptosis is inhibited through the activation of NF-κB p65. It was also reported that mitochondrial dysfunction and dopaminergic neuron loss seen in PD pathogenesis in transgenic mouse models were prevented by quercetin [34].

A study demonstrated the neuroprotective effects of quercetin in traumatic brain injury (TBI) mouse models. Peroxisome proliferator-activated receptor gamma coactivator 1-alpha (PGC-1α) plays crucial roles in the CNS, including stimulating nuclear receptors and their functions. Considering the results of the study, it was observed that the transport of PGC-1α protein from the cytoplasm to the nucleus was substantially accelerated and the nuclear PGC-1α level increased. At the same time, cytochrome c, superoxide dismutase, and malondialdehyde levels in the mitochondria were restored. For these reasons, it was shown that quercetin significantly reduced TBI-induced neuronal apoptosis and increased mitochondrial biogenesis activities [37].

Sirtuins serve a purpose in many molecular processes such as metabolism regulation, cellular stress responses, and longevity. Studies have demonstrated that quercetin has a protective effect against neurodegeneration of dopaminergic neurons by promoting SIRT1 activity [37]. It has also been stated that quercetin contributes to neuroprotection mechanisms by showing antidepressant effects. It has been observed that quercetin promotes adult hippocampal neurogenesis after intragastric administration to mice with depressive-like behaviors induced by chronic unpredictable mild stress. After adult hippocampal neurogenesis, which was stimulated by the Forkhead box transcription factor G1 (FoxG1)/Cyclic AMP-Response Element Binding Protein (CREB)/Brain-derived neurotrophic factor signaling pathway, the abnormal behaviors of the mice were significantly alleviated. Especially, upregulation of CREB, a transcription factor that is continuously expressed in the nucleus and controls the expression of genes involved in neuronal functions, survival, and neurogenesis, strengthens the neuroprotective effect of quercetin [36].

### 2.5. Ginko Biloba Extract

*Ginkgo biloba* (*G. biloba*) is one of the oldest known seed plants. It was the first plant to germinate after the Hiroshima atomic bomb explosion in Japan. *G. biloba* is a very old species and contains many bioactive components such as flavonoids and terpenoids in its structure [39]. The therapeutic effects of these bioactive compounds have been examined in various studies and *G. biloba* extracts have become an important source of new herbal drugs. Studies on *G. biloba* have shown therapeutic benefits in the treatment of asthma, bronchitis, ischemic heart disease, tuberculosis, and diabetes.

*G. biloba* extract (EGb-761) scavenges free radicals produced by ROS and reactive nitrogen species (RNS), and this antioxidant property may have a synergistic effect with other plant extracts. *G. biloba*, which has numerous characteristics such as anticancer, antiplatelet, immunomodulatory, antidepressant, and antimicrobial, in addition to antioxidant, has neuroprotective effects especially on neurological conditions such as tardive dyskinesia, cerebral ischemia, and dementia [38]. The therapeutic effects of EGb-761 on experimental stroke were investigated after induction of ischemia in the mouse brain with middle cerebral artery occlusion. After the administration of EGb-761, it suppressed ischemia-induced glutamate release and reduced excitotoxicity. Thus, the neuroprotective effects of EGb-761 on cellular degeneration were demonstrated [43,142]. Nitric oxide derived from neuronal NOS promotes oxidative damage. Nitrite and nitrate increase was observed in the hippocampus after cerebral ischemia. Nitric oxide formation was significantly decreased after treatment with *G. biloba* extract (Ph-Gb) [43]. Besides its free radical scavenging property, the neuroprotective property of *G. biloba* extract was also studied on PD. The therapeutic effect of EGb-761 treatment on 6-hydroxydopamine-induced lesions in the nigrostriatal dopaminergic system was investigated. It was shown that the progressive deficit in behavioral and motor activity caused by the loss of dopamine neurons in the substantia nigra and the decrease in striatal dopamine after treatment was reduced. [41]. In another study, it has been shown that *G. biloba* affects the neurotransmitter system in autism spectrum disorder, which has no specific treatment. The antioxidant and anti-inflammatory properties of *G. biloba* have been shown to have a neuromodulatory effect on valproic acid-induced autistic pathogenesis. Histopathological results have shown that myelin basic protein and serotonin expression are regulated by *G. biloba* and can be used as a potential treatment for autism [42]. EGb-761 also provides benefits for cognitive impairment caused by vascular dementia (VaD). Although cholinesterase inhibitors are used for the treatment of VaD, there is no effective treatment. It has been observed that cholinesterase inhibitors provide significant cognitive and behavioral benefits on VaD after treatment with EGb-761 [45].

### 2.6. Anthocyanins

Anthocyanins are flavonoids with strong antioxidant, anti-inflammatory, and anti-apoptotic properties. They give blue, red, or purple colors to flowers, fruits, and plants including red cabbage, blueberries, black currants, and mulberries. Anthocyanins are water-soluble and rapidly absorbed after consumption and have neuroprotective effects in the CNS because they can cross the BBB. Studies have demonstrated that cyanidin-3-O-glucoside, the main component of anthocyanins, has positive effects on learning and memory disorders by modulating signaling pathways effective in neurodegeneration. In addition, anthocyanins, which help prevent neurotoxicities caused by different toxic factors such as glutamate accumulation, ethanol, and hydrogen peroxide, also provide neuroprotective effects on neuronal apoptosis [49].

A study conducted to examine the effects of anthocyanins on cognitive behavior has shown that they eliminate cognitive deficits by regulating synaptic plasticity after preventing and clearing the accumulation of age-related toxic proteins [48]. Anthocyanins, which have protective effects against mitochondrial oxidative stress (MOS) due to their antioxidant properties, have been reported to prevent MOS-induced apoptosis by preserving mitochondrial glutathione levels and inducing NRF2 activation [46]. Another study showed that neuronal loss in rats with traumatic spinal cord injury was prevented by anthocyanins. [47]. As a result, anthocyanins have therapeutic and neuroprotective effects in many diseases affecting the CNS.

### 2.7. Luteolin

Luteolin is obtained from plant extracts such as *Terminalia chebula*, *Salvia tomentosa*, and *Matricaria chamomilla*. Luteolin, a yellow crystalline flavonoid, has been used for dyeing purposes since ancient times. Luteolin, which can be obtained through diet from food sources including celery, green tea, olive oil, and broccoli, has antibacterial, antiulcerogenic, and neuroprotective properties [51]. In addition to these properties, it has been widely used in traditional medicine to reduce abdominal pain and heal wounds. A study investigated the anti-inflammatory properties of luteolin purified from the *Pedilanthus tithymaloides* plant. The results demonstrated that it significantly reduced acetic acid-induced pain responses and relieved inflammation [52].

Luteolin provides neuroprotective effects in diseases such as AD, PD, MS, TBI, and stroke. Luteolin crosses the BBB by regulating Rho GTPase, a GTP (guanosine-5′-triphosphate) binding protein involved in intracellular signaling, and inhibits neuroinflammatory responses of microglia and astrocytes by suppressing oxidative stress [51]. Also, the anticonvulsant properties of luteolin, which is used in Ayurvedic medicine to treat epilepsy, have been studied in an electroshock rat model. It has also been reported that luteolin application has anxiolytic properties [52]. Another study on TBI models showed that luteolin increased NRF2-ARE (antioxidant response element) binding and thereby restored malondialdehyde levels and glutathione peroxidase (GPx) activity, significantly ameliorating secondary brain injury induced by TBI [54]. In another study, it was shown that it prevents Aβ accumulation seen in AD pathogenesis by reducing the expression levels of beta-site amyloid precursor protein cutting enzyme 1 (BACE-1) [53].

### 2.8. Apigenin

Apigenin is found in *Hypericum perforatum*, also known as St. John’s wort, as well as *Matricaria chamomilla* and *Melissa officinalis* species. Other plants and natural sources containing apigenin include celery, parsley, and thyme [55]. Apigenin, which is usually found in glycoside form and sometimes as an aglycone, has neuroimmunomodulatory and neuroprotective effects on neurodegenerative conditions [59].

It has been investigated that cognitive function disorders of the mice are eliminated after oral apigenin treatment on APP/PS1 mice, a transgenic mouse model widely used in Alzheimer’s disease research. In addition, it has been reported that BACE-1 levels are downregulated by apigenin, and Aβ accumulation is alleviated [58]. When its therapeutic effects on spinal cord injury are examined, it has been shown that it reduces superoxide dismutase and GPx activity and reverses the increase in malondialdehyde levels due to its antioxidant properties. In addition to this property, it has been shown to have anti-inflammatory effects by reducing IL-1β and TNF-α release [57]. In a study, the gene expression of AMP (adenosine monophosphate)-activated protein kinase (AMPK), which regulates energy homeostasis, was modulated by apigenin. This highlighted that energy production dysfunctions seen in various psychiatric disorders such as depression are eliminated by the anti-depressive properties of apigenin [56].

### 2.9. Fisetin

Fisetin is a hydrophobic and natural bioactive compound. As other polyphenols, it has important biological and functional properties. It is found in strawberries, apples, lotus, cucumbers, tomatoes, and dates. Its pharmacological activities include antioxidant, anti-inflammatory, and neuroprotective properties. For this reason, it is frequently used in pharmacotherapeutic strategies [60].

It has been shown that fisetin treatment in paralyzed patients suppresses the inflammatory response by reducing the production of inflammatory mediators (TNF-α, IL-1β, and NOS) and thus, prevents neuronal dysfunction and death by modulating the activity of inducers that trigger neurodegenerative mechanisms [60]. It has also been shown that treatment with fisetin reduces Aβ accumulation and improves inflammation and synaptic dysfunction for Aβ-injected mice [61]. Excessive increases in glutamate levels or glutamate receptors, which are seen in the pathophysiology of various brain injuries and neurodegenerative diseases, can cause excitotoxicity. Glutamate, which acts as a neurotransmitter in the CNS, plays an important role in synaptic transmission. However, excitotoxicity damages or leads to the death of nerve cells. It is known that fisetin, which has a hydrophobic structure, can protect against exogenous glutamate [60]. Oxidative stress plays an important role in ALS, a progressive neurodegenerative disease associated with superoxide dismutase 1 gene mutation. The antioxidant properties of fisetin showed a neuroprotective effect by helping to maintain redox homeostasis and reduce superoxide dismutase 1 protein. It also increased the expression of antioxidant factors by activating ERK (extracellular signal-regulated kinase). Thus, it has been shown as a potential treatment candidate for ALS [62].

## 3. Vitamins and Vitamin-Like Compound

### 3.1. Vitamin B

Vitamins are micronutrients that the body needs for the proper functioning of metabolism. In addition to strengthening the immune system and preventing infections, they also play an important role in neurogenesis and neuron survival [143]. B vitamins are water-soluble and contribute to cellular functioning by taking part in many catabolic and anabolic reactions in the body. There are eight types of B vitamins known as B1 (thiamine), B2 (riboflavin), B4 (niacin), B5 (pantothenic acid, panthenol), B6 (pyridoxine, pyridoxamine), B7 (biotin), B9 (folic acid, folate), and B12 (cobalamin) [63].

Vitamin B, which plays a role in the synthesis of neurochemicals, has an important effect on brain function. Vitamin B1, in particular, acts as a coenzyme in the glucose metabolism of thiamine diphosphate (ThDP). Thioester benfotiamine (BTF), known as a thiamine precursor, has antioxidant and anti-inflammatory properties in addition to its coenzyme function and has been shown to improve cognitive impairment in AD [144]. Glutamate provides communication between nerve cells by binding to receptors on the surface of the target nerve cell. The electrical signal changes that occur play an important role in cognitive processes such as learning and memory. Therefore, regulation of glutamate release is important for these processes to continue properly. Excessive increase in glutamate release causes significant neuropathological consequences. It is known that vitamins B2, B6, and B12 have neuroprotective effects by preventing excessive glutamate release. Therefore, the B vitamin group has an important role in modulating central glutamatergic neurotransmission [64]. In addition to the role played by vitamin B3 in the synthesis of coenzymes such as NAD (nicotinamide adenine dinucleotide) and NADP and its contribution to energy production, it also has roles in repairing DNA metabolism, reducing oxidative stress, and acting as an antioxidant. In addition to playing a role in the synthesis of many neurotransmitters and steroid hormones, it serves as a substrate for the synthesis of coenzyme A (CoA) [63]. Studies on the effects of vitamin B7 on neuronal health have shown that it has a potential effect in the treatment of tau-mediated neurotoxicity [145]. The functions of vitamins B9 and B12 are interrelated. The mechanism regulated by the conversion of homocysteine to methionine by vitamins B9 and B12 has positive effects on cognitive dysfunction and diseases such as Alzheimer’s, which are caused by high homocysteine levels [63]. As a result, B vitamins have an important role in brain function.

### 3.2. Vitamin C

Vitamin C (ascorbic acid) is water-soluble and cofactor. Inadequate intake of vitamin C, found especially in citrus fruits and green vegetables, leads to the risk of developing scurvy, which is fatal if left untreated. Apart from oxidative stress and inflammatory responses in the body, vitamin C plays a role in many processes such as neural differentiation and regulation of myelin formation [65].

Due to its antioxidant and anti-inflammatory properties, vitamin C is known to provide neuroprotective effects on neurodegenerative diseases such as PD, AD, and stroke. Vitamin C has been shown to modulate microglial responses and astrocyte activation in the PD mouse model. It has been concluded that it reduces the activation of nucleotide-binding oligomerization domain 3 (NLRP3), which is associated with the pathogenesis of many inflammatory diseases, and thus dopaminergic neuronal cell loss. In addition, improvements in the motor functions of mice have been observed [66]. A study examined the therapeutic effects of vitamin C on oxidative stress caused by ethanol, which causes neuronal cell death. It was observed that vitamin C significantly reduced the production of ROS after treatment. It was also stated that it suppressed microglia and astrocytes and attenuated apoptotic neuronal loss by reversing caspases, which are responsible for the formation of various morphological and biochemical changes during apoptosis [67].

Sepsis, which is defined as a life-threatening organ dysfunction, is the body’s excessive response to infections. It is the most common cause of death in intensive care units, and sepsis survivors experience cognitive dysfunctions such as memory and attention issues. It is thought that this condition may increase their susceptibility to neurodegenerative diseases such as dementia in the long term. When the mechanisms of cognitive dysfunction in sepsis patients are examined, neuroinflammatory response, BBB damage, oxidative stress, mitochondrial dysfunction, and neuronal apoptosis in brain tissue are observed. In a study, the neuroprotective effect of vitamin C was investigated on a rat model created with surgical cecal ligation and puncture (CLP). It was suggested that increased levels of TNF-α in the serum of the created rat model activated matrix metalloproteinase-9 (MMP-9) in the brain, which led to increased BBB permeability. However, it has been shown that BBB permeability is significantly improved with high-dose vitamin C treatment. It has been shown to alleviate oxidative stress damage by activating the NRF2 signaling pathway, which up-regulates the expression of numerous antioxidant genes in response to oxidative stress [68]. Another study showed that vitamin C administration significantly improved neurological deficits by reducing transient middle cerebral artery occlusion-induced nitrosative stress [69].

### 3.3. Vitamin D

Vitamin D is synthesized in the skin through a chemical reaction after exposure to sunlight. Vitamin D, which is fat-soluble and a steroid hormone, is found in fish and mushrooms [71]. Vitamin D regulates genes related to cell differentiation and proliferation and plays an important role in immunity, nervous system development, and its functions. It is thought that vitamin D deficiency in children may be an important risk factor for the development of MS in later periods [72]. Vitamin D deficiency is also thought to be strongly associated with COVID-19 complications and increased mortality. Survivors of COVID-19 with vitamin D deficiency can be expected to experience neurological and neuropsychiatric disorders. These disorders include IS, headaches, and neurodegeneration. Vitamin D, along with its immunomodulatory effects, is thought to mitigate the deleterious consequences of COVID-19. It is also known that the inflammatory response seen as a result of overexpression of the vasopressor arm of the renin-angiotensin system is down-regulated by vitamin D [72]. The severity of vitamin D deficiency, which is generally seen in patients after IS, varies according to stroke recurrence and outcomes. Therefore, vitamin D can be considered as a marker for the prediction of survival of patients who have had a stroke. Vitamin D treatment after IS can prevent BBB dysfunction by inhibiting the production of ROS due to its antioxidant properties [146].

IGF-1 and GH (growth hormone) signaling pathways, which are associated with cell proliferation and differentiation, are related to the aging process. Vitamin D is known to be the main regulator of the mechanisms involving IGF-1 and GH. It has been shown to reduce the occurrence of age-related diseases in animal models and contribute to longevity [70]. A study on cognitive impairment in learning and memory caused by scopolamine has shown that vitamin D improves the levels of brain-derived neutrophilic factors. Therefore, vitamin D has a neuroprotective effect with important roles in the CNS and neurodegenerative damage [147].

### 3.4. Vitamin E

There are many studies that show that neuronal cell death is prevented by free radical scavenging antioxidants such as vitamin E. In particular, vitamin E, found in seeds, nuts, vegetable oils, grains, fruits, and vegetables, is known to show the survival of neuronal cells that have suffered glutamate damage by inhibiting glutamate-induced pp60 (c-Src) kinase activation [4]. However, recent studies show that vitamin E has other potential benefits beyond its known antioxidant activity. Vitamin E has 8 forms: α-, β-, γ- and δ-tocopherols and α-, β-, γ- and δ-tocotrienols. Tocotrienols have an unsaturated isoprenoid side chain. Tocopherols have a saturated phyto tail [76]. Studies have shown that δ- and γ-tocotrienol only prevent apoptosis due to oxidative stress, while α-tocotrienol prevents both oxidative stress-induced and oxidative stress-independent apoptosis. In other words, α-tocotrienol has been shown to have anti-apoptotic properties in addition to its antioxidant properties [4]. Another study has reported that vitamin E has an anti-inflammatory effect by suppressing p38 mitogen-activated protein kinase and NF-κB activation. The suppression of these signaling steps associated with microglial activation emphasizes the importance of vitamin E in microglial toxicity and neuroprotection [75].

The orexin system, known as the hypocretin system, consists of a neuron population located at the hypothalamic level and has the function of producing neuropeptides. Neurotransmitters produced from these neurons regulate cholinergic and monoaminergic system activity in the sleep cycle. Problems in the hypocretin system can be a precursor to emotional disorders such as narcolepsy and depression. It is thought that vitamin E plays a role in the activation of the NRF2/ARE pathway, which is seen to be related to the hypocretin system. Hypocretin, which has an ARE region in its promoter, can be activated dependent on NRF2. It has been shown that α-tocopherol, a form of vitamin E, activates NRF2 in neuronal cells and this causes hypocretin to be expressed more. As a result, it is emphasized that vitamin E provides neuroprotection with the formation of many hypocretin-specific peptides in the cells [74].

### 3.5. Vitamin K

Vitamin K, which is found in nature in two main forms as K1 (phylloquinone) and K2 (menaquinone), is a fat-soluble vitamin. Vitamin K1 is found in green leafy vegetables and some vegetable oils. Vitamin K2 has two subtypes: menaquinone-4 (MK-4) and menaquinone-7 (MK-7). MK-4 is obtained from animal foods and is also synthesized by bacteria in the intestine. MK-7 is found in fermented foods [148]. Vitamin K1 plays an important role in blood clotting, while vitamin K2 is effective in bone health.

In addition to these roles, vitamin K has also been shown to have an effect on CNS health. A study showed that antibiotic-induced intestinal dysbiosis in mice led to memory loss. Cognitive dysfunctions observed in mice after vitamin K2 administration were partially reversed by reducing oxidative stress. This study, which shows the relationship between the CNS and the intestinal microbiota, showed increased myeloperoxidase levels in the colon and brain after intestinal dysbiosis. It was concluded that vitamin K2 reduces acetylcholine esterase and other ROS caused by antibiotics due to its antioxidant properties, thus protecting hippocampus neurons [149]. Another study showing that vitamin K is associated with dementia and memory indicated that AD pathology, neurofibrillary tangle density, and Lewy bodies are less likely to be present in brains with high MK-4 concentrations [77].

### 3.6. Coenzyme Q10

Coenzyme Q_10_ (CoQ_10_) is a fat-soluble, vitamin-like essential component of the mitochondrial electron transport chain. CoQ_10_ plays an important role in lysosomal metabolism in addition to mitochondrial oxidative phosphorylation. It can be produced endogenously in the body, as well as taken externally with food or supplements [150]. It is thought that CoQ_10_ deficiency may cause mitochondrial dysfunction. Energy disorders and cognitive and emotional disorders occur in relation to this. Therefore, there are many studies on CoQ_10_ deficiency, which plays a role in the pathophysiology of neuropsychiatric disorders, and its treatment. CoQ_10_ synthesis may be related to genetic deficiency, while its synthesis decreases with age. Some drugs used especially by the elderly such as statins also inhibit CoQ_10_ synthesis [80,81]. Studies have observed significantly decreased CoQ_10_ levels in the blood, plasma, platelet mitochondria, cerebrospinal fluid, and cortex region of PD patients. However, studies on animal models have shown that CoQ_10_ can protect mitochondrial function and the nigrostriatal dopaminergic system. Therefore, it is thought to be a protective agent for Parkinson’s patients [78,79]. Early oxidative stress is seen in HD, a neurodegenerative genetic disease caused by the expansion of CAG repeats in the HD gene. Following mitochondrial dysfunction, striatal neurons appear to die. As a result, symptoms such as dementia and weight loss are observed. As a result of therapeutic studies conducted with CoQ_10_ to improve this neurodegenerative process, it has been shown that oxidative stress markers are reduced. It has also been reported that high doses of CoQ_10_ are safe and tolerable [78].

Doxorubicin (DOX) is a drug used in cancer chemotherapy. However, its neurotoxic effects have been investigated in recent years. It is thought that the activity of acetylcholinesterase (AChE), which is released from nerve endings and stops nerve impulses by hydrolyzing acetylcholine (neurotransmitter), is changed by doxorubicin. When the potential neuroprotective activity of CoQ_10_ against DOX-induced behavioral disorders was investigated, it was shown that AChE activity was inhibited in the brain tissue of rats, and DOX-induced behavioral changes were significantly alleviated [82].

Friedreich’s ataxia (FRDA), an autosomal recessive disease with symptoms including gait disturbance and speech problems, causes progressive damage to the nervous system. Following long-term use of CoQ_10_ and vitamin E in FRDA patients, a permanent improvement in mitochondrial energy synthesis and a decrease in disease progression have been observed. In the treatment of autistic spectrum disorders, which are neuropsychiatric diseases, the combination of CoQ_10_ and B vitamins has also been shown to protect against the progression of oxidative damage and mitochondrial dysfunction [78,79]. This evidence demonstrates the importance of therapeutic pathways and agents in treatment approaches, such as strengthening antioxidant protection, increasing mitochondrial oxidative phosphorylation, and targeting iron chelation.

## 4. Hormones

### 4.1. Melatonin

A neurohormone is a melatonin secreted from the pineal gland and associated with the sleep–wake cycle. It has a wide range of functions in the body, including anti-apoptotic, anti-inflammatory, antioxidant activity, and involvement in the receptor-dependent signaling pathway [84]. In addition, neuroprotective effects on various neurodegenerative diseases have also been demonstrated. Melatonin has been shown to inhibit β-amyloid synthesis and fibril formation, which are seen in the pathogenesis of AD [85]. Thanks to its antioxidant characteristics, it contributes to improving the outcomes of IS by minimizing tissue damage by clearing reactive oxygen species. For this reason, melatonin has crucial potential as a therapeutic agent [83].

### 4.2. Estrogen: Estradiol-17β

Estradiol-17β (E2) is a kind of estrogen and steroid hormone produced in female ovaries and has important functions in female reproduction. Along with its physiological effects such as cell growth and cell differentiation, it also has beneficial effects including preventing bone loss and reducing the risk of coronary disease [83]. In addition, it is known to reduce oxidative stress by protecting the CNS from harmful stimuli and to have neuroprotective effects on ischemic brain injury [87]. It has also been shown to improve cognitive function by increasing hippocampal neurogenesis in AD patients [151].

The important regulatory effects of estradiol-17β on the development and neurotransmission of dopaminergic neurons are realized by the regulation of gene expression through the activation of its own receptors that function as transcriptional factors. In a study, estradiol-17β treatment was applied to weaver mice, a phenocopy of PD. In this phenotype, dopaminergic neurons undergo gradual degeneration. It has been shown to increase neuroprotection of dopaminergic neurons in the substantia nigra (SN) of mice after treatment with estradiol-17β [86].

## 5. Neurotransmitters

### 5.1. Serotonin and Selective Serotonin Reuptake Inhibitors (SSRIs)

Serotonin is an important neurotransmitter in the CNS. As a hormone, it controls various physiological functions. Its physiological functions include the control of mood, anxiety, sleep, and intestinal motility. Serotonin, which reduces oxidative stress and ensures the survival of nerve cells, has neuroprotective effects due to its antioxidant properties. Serotonin is synthesized from tryptophan by tryptophan hydroxylase (TPH) [92]. Adequate dietary tryptophan intake from foods such as chicken, eggs, dairy products, bananas, and walnuts can support serotonin synthesis [152].

Dysfunction of the serotonergic system, which includes the production, release, uptake, and function of serotonin, can cause conditions such as depression and other mood disorders, schizophrenia, and autism [91]. Selective serotonin reuptake inhibitors, which are among the synthetically produced drugs, help increase the amount of serotonin in the synaptic cleft by preventing the reuptake of serotonin. The increase in serotonin levels helps relieve symptoms of mood such as depression and anxiety. In addition to their neuromodulatory roles, when their potential effectiveness on COVID-19 is examined, SSRIs have been shown to bind to serotonin transporters and σ-1 receptors, thus relieving inflammation, and their immunomodulatory properties have also been shown [90]. In addition to anxiety seen with depression, obsessive–compulsive symptoms can also be seen together. It is known that SSRIs and serotonin and norepinephrine reuptake inhibitors (SNRIs) used as treatment relieve these symptoms, but clinical research results are available for short-term use. There are not enough data on long-term SSRI use [88,89]. A study conducted on this subject showed that the results of SSRI use for more than 24 months showed that long-term SSRI use prevents relapse. It was stated that in the event of discontinuation of SSRI use, there may be a risk of relapse in the disease, permanent disorder, or unresponsiveness to interventions, along with recurrence of symptoms [88].

### 5.2. Dopamine and Agonists

Another neurotransmitter that has important roles in the CNS and is synthesized from tyrosine is dopamine. Tyrosine can be obtained from a diet of nuts, legumes, meat, fish, and dairy products [153]. Adequate intake of tyrosine can support dopamine synthesis and release in nigrostriatal neurons [154].

Dopamine, which is associated with motor control, reward mechanisms, motivation, and cognitive function, also supports the survival of nerve cells [95]. Dysfunction of the dopaminergic system, which includes the production, release, uptake, and function of dopamine, is seen in many neurological and psychiatric disorders. Dopamine receptors (D1, D2, D3, D4, D5) are found on cell surfaces where dopamine binds and signal transmission occurs. It is thought that the D2 receptor, in particular, may have neuroprotective effects in diseases such as epilepsy and ischemia [97]. Dysfunctions of the dopaminergic system can cause increased oxidative stress, inflammation, protein misfolding, aggregation, and apoptosis [155].

Free radicals oxidize dopamine; therefore, dopamine agonists, such as levodopa improve the oxidative damage seen in dopaminergic neurons. It is also known that levodopa is used in the symptomatic treatment of PD by preventing motor complications. Other studies have shown that other dopamine agonists have neuroprotective effects such as reducing dihydroxybenzoic acid (DHBA) efflux and improving the lesion induced by 1-methyl-4-phenylpyridinium (MPP+), stabilizing the ubiquitin–proteasome system, and promoting the synthesis of growth factors and neurogenesis [50,96].

### 5.3. Gamma-Aminobutyric Acid (GABA) and Agonists

Gamma-aminobutyric acid (GABA) is an amino acid and neurotransmitter synthesized from glutamate by glutamate decarboxylase (GAD). Cells, receptors, and signaling pathways associated with GABA constitute approximately 60–75% of the brain. Therefore, GABA plays an important role in the CNS [98]. GABA cannot be obtained directly from the diet, but the GABA precursor glutamate is found in protein-rich foods. Low levels of glutamate support the growth and plasticity of nerve cells, while high levels of glutamate can lead to excitotoxicity. However, green tea and fermented foods are known to increase GABA levels [156,157].

GABA, which has physiological functions such as antihypertensive and antidepressant, has effects such as strengthening immunity, reducing anxiety, improving insomnia, and reducing stress. A study has shown that the released GABA suppresses the proinflammatory response in astrocytes and microglia caused by Aβ accumulation [158]. In another study, it was shown that the decreases in GABAergic neuronal density seen in the pathogenesis of schizophrenia, abnormalities in receptors, and reuptake areas were corrected with GABA agonists, and thus the cognitive symptoms of schizophrenia patients could be improved. When the treatment mechanism is examined, it is seen that the abnormalities occurring in the mesolimbic dopaminergic system are related to symptoms such as hallucinations and delusions, and the disorder occurring in the GABAergic system that regulates this system is improved with GABA agonists [99].

### 5.4. Acetylcholine

Acetylcholine (ACh) is synthesized from choline by the enzyme acetyltransferase at the ends of cholinergic nerve cells. Acetylcholine, which controls skeletal muscle and the autonomic nervous system, is stored in vesicles until stimulated after being synthesized [100].

Cholinergic signaling and ACh are known to play a role in attention, memory, and learning. Acetylcholine levels are reduced in AD. ACh protects nerve cells and has a neuroprotective effect against neurodegenerative diseases [101]. Acetylcholine shows its effects by binding to two main types of receptors: nicotinic acetylcholine receptor (nAChR) and muscarinic acetylcholine receptor (mAChR). Pharmacological studies show that both receptors play a role in the coding of new memories [102]. Reflecting the characteristic feature of AD, Aβ peptide directly communicates with nAChRs. Therefore, it can be used as a treatment for AD by showing both agonist and antagonist effects on nAChRs [103]. Cholinergic dysfunctions are also known to affect mood states such as depression. Changes in hippocampal neurogenesis after cognitive impairment seen in depression are corrected with cholinergic drugs and provide therapeutic effects [159].

### 5.5. Adrenaline and Noradrenaline

Adrenaline (epinephrine) and noradrenaline (norepinephrine) are neurotransmitters produced by the nervous system. Unlike noradrenaline, adrenaline contains a methyl group. Adrenaline is produced in the adrenal glands and released into the blood. Noradrenaline is produced and released by both the adrenal glands and neurons in the locus coeruleus (LC). The important components of the sympathetic nervous system for stress and emergencies in the body have important physiological functions such as regulating blood pressure and glucose levels, vasoconstriction, and cardiac stimulation.

In addition, noradrenaline plays a role in motor and mental functions, affecting memory, movement, and attention. It also plays a role in neuroplasticity by affecting the differentiation and flexibility of neurons [94]. In a study, degeneration was triggered by exposing cholinergic neurons to oxidative stress in the brains of rats. It was later observed that ROS and proapoptotic caspases were blocked after treatment of these mouse models with noradrenaline. Therefore, it was concluded that noradrenaline-based treatment strategies may be important in cases such as AD, where cholinergic deficiencies are seen [160].

However, it is thought that noradrenaline has a neuroprotective effect by controlling glial activation and chemokine production due to its anti-inflammatory properties. It is thought that maintaining noradrenaline levels in the CNS will help prevent or delay the development of AD. In particular, the use of noradrenaline-regulating drugs is seen as an alternative treatment for AD [161].

## 6. Aminoacids and Its Derivatives

### 6.1. Creatine

Creatine synthesis involves three amino acids (glycine, arginine, and methionine) and two enzymes (transamidinase and transmethylation). Creatine is synthesized by the liver, kidneys, and pancreas [104]. Creatine can be transported in the blood and pass through the BBB, but this process is slow, also synthesized in the body and obtained from animal foods through the diet. Creatine supplementation is especially important for athletes because when the body demands ATP quickly and highly, creatine is converted to phosphocreatine by creatine kinase, supporting ATP production [106].

In addition to these properties, creatine and its analogs have been shown to have antitumor, antidiabetic, and antiviral effects. When its neuroprotective effects are examined, it has been seen that it protects against hypoxic–ischemic, and neurodegenerative damage [108]. Creatine has shown neuroprotective effects by reducing the loss of dopaminergic neurons, especially against the toxicity of MPTP (1-methyl-4-phenyl-1,2,3,6-tetrahydropyridine), a neurotoxin that produces symptoms similar to PD. In ALS transgenic mouse models, it has been reported to improve motor performance, protect motor neurons, and prolong survival. In a study on HD, creatine administration was delayed until the onset of symptoms. Nevertheless, creatine administration improved motor functions by reducing striatal atrophy [105,107].

### 6.2. N-Acetyl Cysteine

*N*-acetyl cysteine (NAC) is a precursor of L-cysteine and a powerful antioxidant. Cysteine, which can be obtained from diets such as chicken, turkey, dairy products, eggs, and garlic, exhibits antioxidant properties such as helping to scavenge ROS and having therapeutic effects for functional disorders caused by free radicals [111]. The level of cysteine, one of the three amino acids used in the synthesis of glutathione, is increased by NAC, promoting glutathione production, and thus increasing glutathione levels also reduces oxidative stress [110,111]. It has been demonstrated that the decrease in glutathione levels seen in mood swings including depression and bipolar disorder can be improved by reducing the symptoms related to the disease after treatment with NAC [109].

### 6.3. Activated Protein C

Protein C, which modulates blood clotting by controlling the anticoagulant pathway, also plays a role in inflammatory responses and endothelial cell apoptosis. The active form of protein C, which is activated through the interaction of thrombin and thrombomodulin (TM), maintains blood fluidity by preventing excessive clotting. Thrombin converts fibrinogen to fibrin and enables the formation of blood clots. Also, it interacts with thrombomodulin and activates protein C from its zymogenic form with the complex it forms. The endothelial cell protein C receptor binds to activated protein C, increasing its activation by the thrombin–thrombomodulin complex by 20 times. Thus, activated protein C inactivates factor Va and factor VIIIa, preventing blood clots from forming [162].

Additionally, the cytoprotective characteristics of activated protein C, such as anti-inflammatory, anti-apoptotic, and endothelial barrier stabilization, seen in endotoxemia and sepsis mouse models, have also been reported to have neuroprotective effects in IS models [112]. In a study, 3K3A-APC, a genetically modified variant of active protein C in an activated protein C mutant with reduced anticoagulant activity, showed cytoprotective effects in neuronal damage. Also, it was reported to have neuroprotective effects by protecting brain endothelial cells from apoptosis caused by oxygen–glucose deprivation [113].

Bax (Bcl-2-associated X protein) and Bcl-2 (B-cell lymphoma 2) play important roles in the apoptosis process. Bax promotes apoptosis while Bcl-2 prevents apoptosis. In a study, activated protein C was shown to regulate the Bax/Bcl-2 ratio by inhibiting the tumor suppressor protein p53 and preventing apoptosis in hypoxic human brain endothelium by reducing caspase-3 signaling [114].

## 7. Proteins

### 7.1. Brain-Derived Neurotrophic Factor (BDNF)

The BDNF gene is located on chromosome 11 in humans and encodes the BDNF protein. BDNF is synthesized from a precursor protein, pro-BDNF, after transcription. BDNF gene expression varies depending on many factors. It has been shown that BDNF mRNA expression increases with light stimulation in the visual cortex, osmotic stimulation in the hypothalamus, electrical stimulation that causes long-term potentiation in the hippocampus, and exercise [118]. The TrkB (tropomyosin receptor kinase B) receptor, to which neutrophic factors bind, plays an important role in the survival and plasticity of nerve cells. It is thought that the increase in BDNF and TrkB receptor activation is effective in various memory processes and learning. It has been shown that induction of the BDNF expression in mutant mouse models where synaptic plasticity is impaired restores plasticity ability [117].

Defects in nerve regeneration not only disrupt structural integrity and lead to abnormalities but also impair the individual’s ability to manage crisis situations. Therefore, it is thought that BDNF also has roles in depression and antidepressant mechanisms. In a study, BDNF and TrkB expression were shown to play a role in the pathophysiology of suicidal behavior, in addition to cell survival and synaptic plasticity. It has been shown that there is a significant decrease in mRNA levels of BDNF and TrkB in the hippocampus and prefrontal cortex of subjects who died by suicide [120].

The symptoms seen in various neurodegenerative diseases such as AD, PD, and HD have been associated with a decrease in BDNF levels and changes in signaling pathways. It is thought that BDNF promoter methylation, BDNF/TrkB levels, BDNF-AS (BDNF antisense RNA), and TrkB isoforms can be used as biomarkers for early diagnosis and risk determination in neurodegenerative diseases where the deficiency of BDNF and TrkB signaling is thought to play a role. It is also emphasized that it can be used as a therapeutic treatment method for neurodegenerative diseases [119]. In addition to these methods, it has been stated that flavonoids that can stimulate BDNF-related signaling have important effects in the presymptomatic stage in treatment methods [122].

A neuropathy called glaucoma occurs as a result of degeneration of optic nerve fibers and apoptotic loss of retinal ganglion cells. This process occurs with vascular irregularity and high intraocular pressure. After the injury, the system that transmits signals from the retina to the brain is disrupted. Survival of retinal ganglion cells is promoted by BDNF and is thought that it can be used as a therapeutic agent in glaucoma neuropathy due to its neuroprotective properties [121].

### 7.2. Glial Cell-Derived Neurotrophic Factor (GDNF)

The GDNF protein encoded by the GDNF gene plays an important role in the CNS, such as promoting the survival of dopaminergic neurons and maintaining many types of neurons. It is considered a promising therapeutic agent for the treatment of neurodegenerative diseases such as PD due to its ability to improve motor disorders and restore the nigrostriatal pathway [123,125].

Traumatic spinal cord injury leads to disruption of the blood–spinal cord barrier after mechanical damage. In the continuation of this process, ischemia, excitotoxicity, edema, and a toxic lesion that prevents axon regeneration can be seen. Astrocytic glial scar formation is observed after damage. It is thought that GDNF has positive effects on astrocytosis, which is the abnormal proliferation or growth of astrocyte cells [125]. In addition, the Akt (protein kinase B)/PI3K (phosphoinositide 3-kinase) signaling pathway was activated by GDNF, inhibiting the mitochondrial-dependent cell apoptosis signaling pathway, thus, demonstrating a protective effect on glutamate-induced cytotoxicity [127]. It has been reported that motor deficits and damaged brain tissues following acute focal cerebral ischemia (FCI) injury in mice were improved by GDNF [126]. In another study on cerebral ischemia, lactate dehydrogenase (LDH) leakage, mitochondrial membrane potential (MMP), and apoptosis processes were examined. The neuroprotective effect in the CNS was demonstrated by regulating MEK (MAP Kinase Kinase)/ERK and PI3K/AKT signaling by stimulating GDNF production [115].

Chronic stress predisposes individuals to psychiatric disorders. GDNF expression is also affected by stress factors. It plays an important role in the pathogenesis of mood disorders such as bipolar affective disorder and depression. Glucocorticoids, known as stress hormones, suppress GDNF expression. The decrease in GDNF expression in the hippocampus of rats that showed signs of depression due to exposure to stress was treated with exogenous GDNF. As a result, it was stated that it has neuroprotective effects by strengthening DA function with an increase in the number of DA neurons [124].

## 8. Others

### 8.1. Omega-3 Fatty Acids

Omega-3 fatty acids can be obtained through a diet of foods such as oily fish, flaxseed, and walnuts. Omega-2 fatty acids, which have important effects on human health, are a member of the polyunsaturated fatty acid (PUFA) group. EPA (eicosapentaenoic acid) and DHA (docosahexaenoic acid) are known as two important components of the omega-3 fatty acid family. EPA, known to be effective in heart health, blood pressure regulation, and the treatment of rheumatoid arthritis, has anti-inflammatory, antiarrhythmic, and antithrombotic properties [163].

DHA regulates brain functions, cognitive development, and sleep cycles. It also has important therapeutic effects in the treatment of mood swings and neuropsychiatric disorders such as depression and schizophrenia. DHA, which protects the structural and functional integrity of the CNS, has important neuroprotective effects [128]. The results of studies conducted on MS pathology have shown a decrease in DHA levels. The decrease in DHA caused a decrease in antioxidant defense. Therefore, ROS increased, and irregularity occurred in the glutathione redox system [164]. As in MS, a decrease in DHA levels has been observed in schizophrenia patients. DHA, which modulates the dopaminergic mechanism, has been shown to reduce oxidative stress and have positive effects on neuropsychiatric disorders when administered to schizophrenia patients [130]. In addition, it has been stated that both EPA and DHA have antidepressant effects due to their anti-inflammatory effects and contribution to neuroplasticity [129].

### 8.2. N-Glycans

Advances in glycobiology have shown that carbohydrates are not only used as an energy source but also play a role in many biological roles such as immune response, signal transduction, and apoptosis [10]. Glycan structures, in which many monosaccharides are linked by glycosidic bonds to form oligosaccharides, play roles in the development of the microbiome, antimicrobial effect, or as a prebiotic [133,134]. Abnormalities observed in the intestinal microbiota can negatively affect the gut–brain axis by altering lipid homeostasis. Therefore, neuronal signaling disorders and the pathophysiology of neurodegenerative diseases such as AD can be observed [7]. The effect of *N*-glycans on the development and progression of AD, where Aβ accumulation is seen, can be both positive and negative. Although previous studies emphasized that *N*-glycans contribute to Aβ accumulation, the neuroprotective effects of other glycan types on AD have been ignored. Therefore, studies on the development of glycan-based AD therapeutics have not progressed [165]. It has been reported that *N*-glycan branching in neurons has a neuroprotective effect on MS pathology such as spontaneous inflammatory demyelination, T cell hyperactivity, and cytotoxic T lymphocyte antigen 4 (CTLA-4) endocytosis. It is thought that GlcNAc (*N*-Acetylglucosamine) branching, especially in neurons, has effects such as preventing apoptosis and suppressing the inflammatory response [131].

One of the most important types of oligosaccharide modifications, the fucosylation process, is carried out by binding the fucose molecule to oligosaccharides with glycosidic bonds. Among the important biological roles of fucosylated oligosaccharides are neuroprotective effects such as supporting neurite migration, neurite growth, and synapse formation [10]. A study showed that L-fucose has a neuroprotective effect by promoting neurogenesis [132]. Therefore, glycobiology is becoming an important field for developing glycan-based drugs and using glycans as biomarkers in therapeutic approaches for neurodegenerative diseases.

### 8.3. Cannabidiol

Cannabidiol (CBD), a component of the cannabis plant, has many pharmacological effects. Due to its lipophilic properties, it is rapidly distributed from the blood to the brain and other organs of the body. CBD is widely used in medical treatments due to its anticonvulsant, and antiepileptic properties, and its benefits in reducing refractory seizures. Studies examining its effects in many neurological and psychiatric diseases have identified more than 65 molecular targets of CBD. At the same time, as a result of these studies, CBD has been shown to have antidepressant and anxiety-reducing properties and to relieve fear associated with trauma [135,137].

Cannabidiol has neuroprotective effects against neurodegenerative diseases such as AD and PD due to its anti-inflammatory, antioxidant, and protein aggregation-reducing properties. When the effect of cannabidiol was examined on autoimmune encephalomyelitis mouse models, CBD showed anti-apoptotic effects by inhibiting ERK phosphorylation and caspase-3 activation and modulating Bax/Bcl2 [136]. Another neuroprotective effect has been shown to reduce the effects of ischemia-induced motor and sensory disorders [138]. In another study, rat brains were stimulated with heroin. After stimulation, changes occur in the glutamatergic system receptors in the brain due to chronic use and addiction. These changes cause impairments in cognitive functions. It has been shown that CBD treatment alleviates opioid withdrawal and normalizes changes in glutamatergic receptors [135].

## 9. Food Sources and Daily Intake Recommendation for Neuroprotective Agents

Polyphenols are naturally occurring compounds found in many food sources, especially fruits and plants. Due to their positive effects on health, these therapeutic molecules are used in the production of functional foods. In addition to polyphenol-enriched food products, they can also be formulated as pharmaceutical drugs or nutraceuticals. However, excessive consumption should be avoided to prevent the accumulation of excess amounts of these molecules in the body [15]. It has been stated that daily polyphenol intake is approximately 1 g/day. It is thought that fruits such as apples, grapes, pears, and cherries contain approximately 200–300 mg of polyphenols in 100 g, and a glass of red wine and a cup of coffee/tea contain approximately 100 mg of polyphenols [166]. Daily dietary habits vary according to geographical conditions. A study estimated that the Italian population consumes an average of 660 mg of polyphenols per day from nuts, cherries, tea, coffee, chocolate, and citrus fruits [167]. Curcumin, another compound used as a dietary supplement, is authorized for use in food supplements in Brazil and the daily consumption amount for individuals over the age of 19 is recommended as 80–130 mg [168]. Resveratrol, which has been studied for its neuroprotective effects, is abundantly found in food sources such as grapes and wine. Resveratrol content is higher in red wine than in white wine. It is known that resveratrol content is also related to fermentation time. In addition to these foods, strawberries, blueberries, peanuts, and mulberries can be given. Due to its pharmacological effects, functional foods and supplements containing resveratrol are available on the market and moderate doses are considered safe. The effective dose is stated to be 30 mg/day [169]. Some of the studies that focused on the toxicity of polyphenol consumption examined the safety of green tea extracts, stating that regular consumption in the traditional infusion form appears to be safe, but that more studies are needed to ensure the safe use of concentrated extracts [15]. However, they concluded that a daily dose of 800 mg for 4 weeks was well tolerated [170]. Another therapeutic molecule that is quite commonly obtained from diets is the approximately 350 different quercetin conjugates identified in plant compounds. The amount of quercetin varies between 10 mg and 500 mg depending on the type of food, and it is known that the highest quercetin content is in *Capparis spinosa* (233.84 mg/100 g). In addition, there are significant amounts of quercetin such as 70.37 mg/100 g in raw radish leaves, 66.19 mg/100 g in raw wild arugula, 55.15 mg/100 g in fresh dill, 52.90 mg/100 g in raw coriander leaves, 48.80 mg/100 g in raw fennel leaves, and 42.00 mg/100 g in fresh Mexican oregano [171]. *G. biloba* leaf extracts, one of the medicinal plants in high demand worldwide, are sold on the market as herbal medicines and dietary supplements. Studies have shown that this extract does not cause any side effects at a dose of 120 mg/kg/day [172]. Consumption of anthocyanins, which are abundant in nature, especially in red, blue, and purple fruits/vegetables, is more common in regions with a Mediterranean diet [173]. Anthocyanin contents in foods have been reported as 400–1500 mg/100 g in black chokeberry, blueberry (60–300 mg/100 g), blackcurrant (100–500 mg/100 g), blackberry (50–350 mg/100 g), and purple corn (≥1500 mg/100 g) [174]. Since anthocyanins are generally not found in daily foods, studies have been conducted on the recommendation for daily consumption of these beneficial compounds. In order to reduce the risks of degenerative diseases as well as diabetes, cancer, and metabolic syndrome, a daily intake of 50 mg has been recommended. When we look at the per capita consumption rates of these compounds worldwide, it was determined as the United States (12.5 mg), Australia (24.2 mg), and Asia (37 mg) [173]. Another polyphenol source found in plants and having neuroprotective effects is apigenin. It is found in nature in plants such as celery, kumquat, parsley, onion, tea, dried thyme, coriander, and vine spinach. The average daily consumption amount was determined to be 3 ± 1 mg/day in Europe, 4.23 mg/day in China, and 0.45 mg/day in Australia [175].

Preventing vitamin deficiency and toxicity, which constitute an important part of diets in terms of health, is another important issue. The daily recommended intake of vitamin C, which is found in high amounts in foods such as asparagus, celery, zucchini, raspberry, Brussels sprouts, bell pepper, broccoli, peas, orange, lemon, and grapefruit, is recommended as 120 mg. The daily recommended intake of vitamin E, which is found in high levels in avocado, spinach, hazelnuts, eggs, milk, and cereals, is 15 mg [166]. The body’s requirement for vitamin B, which is found in dairy products, meat, eggs, fruits, and green vegetables, varies according to the person’s life stage and the types of B group vitamins [176]. For adults, the recommended daily intake of vitamin B1 is 1.1–1.2 mg. The recommended daily intake of B6 for adults is 1.3–1.7 mg. The adequate daily requirement for vitamin B7 has been determined as 35 μg for infants and 150–300 μg for adults, and it has been concluded that doses greater than 60 mg per day for several months are relatively non-toxic. A daily intake of 4 μg of vitamin B12 is thought to be sufficient to maintain biological functions [177]. The most important dietary sources of vitamin D are known to be oily fish and egg yolks. A daily vitamin D intake of 400–800 IU is recommended. However, consumption of these foods is not common in some regions. For this reason, vitamin D is generally consumed as a food supplement by some countries [178,179]. Vitamin K, which is generally found in photosynthetic plants, is mostly found in vegetables with high chlorophyll concentrations such as kale and spinach [180]. The recommended adequate daily intake of vitamin K aims to ensure normal blood clotting. However, studies conducted so far also target bone and vascular health. For this reason, it is recommended that more than 250 μg of vitamin K intake is necessary [181]. Coenzyme Q10, the third most consumed food supplement after fish oil and multivitamins, has a wide distribution in plant and animal tissues. Since Coenzyme Q10 is distributed in high-energy tissues, animal hearts and livers represent the richest dietary source (30–200 mg/kg). In meat, fish, and nuts, this rate is known to be 10–50 mg/kg, and in grains and fruits/vegetables, this rate is known to be approximately 1–10 mg/kg [182]. Another therapeutic hormone, melatonin, is a compound naturally secreted by the body. Studies conducted on melatonin in the treatment of diseases such as obesity and hypertension have shown that melatonin supplementation has positive effects on the treatment strategies of these diseases. In obese women, daily 6 mg melatonin supplementation before bedtime has been shown to indicate improvements in inflammatory parameters such as tumor necrosis factor-alpha and interleukin-6. When looking at natural dietary sources, melatonin was found in concentrations in chicken heart and liver mixture (1.1 ± 0.01 ng/g), lamb (1.6 ± 0.14 ng/g), chicken with skin (2.3 ± 0.23 ng/g), pork (2.5 ± 0.18 ng/g), beef (2.1 ± 0.13 ng/g), salmon (3.7 ± 0.21 ng/g), and dried egg powder (6.1 ± 0.95 ng/g) [183]. A diet rich in tryptophan has been associated with higher levels of neurotransmitter serotonin. The recommended daily requirement of tryptophan, which can be found in foods such as chicken, eggs, dairy products, bananas, and walnuts, is 900–1000 mg [184]. Tyrosine, found in nuts, legumes, meat, fish, and dairy products, also plays a role in neurotransmitter synthesis and regulation. Tyrosine intake increases dopamine synthesis and release in nigrostriatal neurons. Another neurotransmitter produced depending on the amount of tyrosine is norepinephrine. In addition, vitamin B, vitamin C, vitamin D valine, isoleucine, leucine, phenylalanine, iron, zinc, and omega-3 fatty acids also increase the synthesis of other neurotransmitters. Tea, a source of theanine, is thought to increase GABA levels in particular. There are many foods that contain acetylcholine, known as the main neurotransmitter in the brain. Examples include turkey, peas, salmon, egg yolk, mung bean, soybean, beef, and lentils. However, the highest concentration of acetylcholine is found in nettles. Mistletoe, a type of nettle, has traditionally been used to treat patients with high blood pressure, headaches, epilepsy, and other neurological disorders. It is thought that the suppressive and calming effects of mistletoe on the heart may be due to the presence of GABA in addition to the acetylcholine it contains [185]. The daily requirement for creatine, an amino acid and its derivatives, is 2–2.5 g. Half of this is synthesized endogenously by the kidney, liver, and pancreas, while the other half is taken into the body through an omnivorous diet [186]. While plant-based foods from natural food sources do not contain creatine, it is found in animal foods. In particular, creatine can be found in breast milk at 0.2 g/kg and up to 11.0 g/kg in herring filet [187]. Cysteine deficiency seen in people as they age causes a loss of quality of life. Daily intake of less than 1200 mg of cysteine, which is naturally found in fish, meat, milk, eggs, and soybeans, may not show significant benefit [188]. Due to its rapid intestinal absorption and metabolism, cysteine is generally known to be safe and well tolerated even in high doses [189]. Protein C, which has neuroprotective effects, is a vitamin K-dependent glycoprotein and is not found in foods or nutraceuticals. Therefore, protein C synthesis is dependent on vitamin K levels [190]. Endogenously produced BDNF plays a critical role in brain health, including neuroprotection, neurogenesis, and synaptic plasticity. Dietary strategies to increase the levels of these proteins in the body, which cannot be supplemented orally, are important. In particular, it is known that α-linolenic acid intake increases BDNF expression in the body. Another component that increases BDNF levels and is found largely in fish, flaxseed, and walnuts is omega-3 fatty acid [191,192]. Likewise, there are indications that polyphenols have a positive effect on BDNF and GDNF concentrations [193].

## 10. Conclusions

The brain contains many interconnected and complex cellular and molecular mechanisms. Brain damage can cause permanent dysfunctions in these mechanisms. The brain continues to be an area where surgical intervention cannot be performed except for the removal of tumors and the repair of some aneurysms. Some agents that can cross the blood–brain barrier and reach neurons show neuroprotective effects in the brain due to their anti-apoptotic, anti-inflammatory, and antioxidant properties. In particular, some neuroprotective agents act by reducing or modulating the accumulation of protein aggregates in neurodegenerative diseases (Alzheimer’s disease, Parkinson’s disease, Huntington’s disease, Amyotrophic lateral sclerosis, prion disease) caused by protein accumulation. Trauma, IS or hypertension can disrupt the blood–brain barrier. With the increase in the permeability of the blood–brain barrier, the entry of harmful substances into the brain tissue increases. As a result, communication between nerve cells is disrupted and damage occurs in motor and cognitive functions. Exposure to toxins or chemicals or age-related metabolic diseases causes dysfunction in mitochondria. Dysfunction of mitochondria results in the accumulation of ROS and increased oxidative stress. As a result, neuroinflammation is observed in the brain. Microglia and astrocytes, which are the immune cells of the brain, produce an inflammatory response by releasing proinflammatory cytokines such as IL-1β, TNF-α, and IL-6. Cytokines released with long-term or chronic inflammation can trigger apoptosis. Brain cells are damaged and cannot function after neuronal apoptosis. PI3K, Akt, GSK3β, Bax, Bcl-2, and caspase-3, which play a role in apoptosis, are important molecules that play a role in cell signaling and apoptosis. Neuroprotective agents modulate apoptosis processes by acting in the activation and inhibition of these molecules. It also plays a role in neuroplasticity, known as self-restructuring and brain adaptation. In addition, it can support neurogenesis by promoting cell proliferation after neuronal damage. Due to the important roles that neuroprotective agents play in CNS damage or neurodegenerative diseases, it is important to elucidate many mechanisms. The flavonoid group, which is found in natural sources, foods, and plants and can be taken with the diet, constitutes a large part of these bioactive compounds. There are many studies on the neuroprotective effect of flavonoids and the mechanisms by which they act. In addition to these studies, both in vivo and in vitro studies are ongoing on the neuroprotective effect of vitamins, neurotransmitters, hormones, amino acids, and their derivatives and pharmacological approaches to develop neuroprotective agents are gaining importance. The development of therapeutic agents that target a single cascade in multiple molecular mechanisms involved in brain damage may not always be an effective treatment method. For this reason, focus is being placed on multiple therapeutic agents that can target synergistic pathways. However, there are challenges such as identifying such agents and determining their toxicity. In addition, combined nanoparticle designs are of great interest to increase the potential interaction between functional nutrients and neuroprotective agents and to enable these agents to cross the blood–brain barrier and reach the target region. However, emphasis should be placed on studies examining the long-term effects of diets or supplements that are already available in nature or on the market. Current studies focusing on the complex and mutually reinforcing mechanisms of neuroprotective agents show that neuroprotective agents that cannot be taken exogenously and can only be synthesized in the body are related to the levels of other agents taken externally through diet. Therefore, further studies are needed on the relationship between various other nutraceuticals and non-exogenous agents. Consequently, understanding the mechanisms of neuroprotective agents contributes to the development of pharmacological drugs and the use of new therapeutic agents, thus enabling new treatment strategies.

## Figures and Tables

**Table 1:** Potential benefits and action mechanisms of neuroprotective agents.

Neuroprotective Agents	Compounds	Action Mechanisms	Potential Benefits	References
Polyphenols	Curcumin	Anti-inflammatory, immunomodulator, antioxidant	Protecting substantia nigra neurons, treating spinal cord injuries, and reducing the risk of AD and PD.	[16,17,18,19,20,21]
	Resveratrol	Antioxidant, anti-inflammatory, anti-apoptotic, inhibits Aβ accumulation	Regulating mitochondrial function, improving balance and motor coordination by promoting remyelination, and protecting neurodegeneration.	[22,23,24,25,26,27,28]
	Green tea polyphenols	Antioxidant, anti-inflammatory, anti-apoptotic, inhibits Aβ accumulation	Protecting spinal cord injuries and AD pathogenesis, improving cognitive functions, and suppressing the expression of pro-apoptotic genes.	[29,30,31,32]
	Quercetin	Antioxidant, anti-inflammatory, inhibits Aβ accumulation, anti-depressant	Increasing neurogenesis, preventing dopaminergic neuron loss, and restorating mitochondria.	[26,33,34,35,36,37]
	*Ginko biloba* extract	Immunomodulatory, antioxidant, antidepressant, anti-inflammatory	Improving cognitive and behavioral function, reducing glutamate excitotoxicity.	[7,38,39,40,41,42,43,44,45]
	Anthocyanins	Antioxidant, anti-inflammatory, anti-apoptotic	Preventing neurotoxicities and neuronal loss, regulating synaptic plasticity.	[46,47,48,49,50]
	Luteolin	Anti-inflammatory, anticonvulsant, anxiolytic, inhibits Aβ accumulation	Treating epilepsy, improving behavioral function, protecting AD pathogenesis.	[51,52,53,54]
	Apigenin	Immunomodulatory, anti-inflammatory, inhibits Aβ accumulation, antioxidant, antidepressant	Protecting AD pathogenesis, providing energy homeostasis, improving psychiatric disorders.	[55,56,57,58,59]
	Fisetin	Anti-inflammatory, antioxidant, inhibits Aβ accumulation	Preventing neuronal dysfunction and death, protecting AD pathogenesis, and reducing glutamate excitotoxicity.	[60,61,62]
Vitamins and vitamin-like compounds	Vitamin B	Anti-inflammatory, antioxidant	Improving cognitive impairment, treating the tau-mediated neurotoxicity.	[63,64]
	Vitamin C	Anti-inflammatory, antioxidant, anti-apoptotic	Providing neural differentiation and myelin formation, improving motor functions.	[65,66,67,68,69]
	Vitamin D	Anti-inflammatory, Immunomodulatory, antioxidant	Regulating cell differentiation and proliferation, providing longevity, and improving cognitive functions.	[70,71,72,73]
	Vitamin E	Antioxidant, anti-apoptotic, anti-inflammatory, antidepressant	Protecting microglial toxicity, improving emotional disorders.	[4,74,75,76]
	Vitamin K	Antioxidant	Protecting dementia, improving memory, and preventing intestinal dysbiosis.	[77]
	Coenzyme Q_10_	Antioxidant	Improving emotional disorders, protecting mitochondrial function and nigrostriatal dopaminergic system, protecting PD, improving cognitive functions, and treating autistic spectrum disorder.	[78,79,80,81,82]
Hormones	Melatonin	Anti-apoptotic, anti-inflammatory, antioxidant, inhibits Aβ accumulation	Improving the outcomes of ischemic stroke, protecting AD pathogenesis.	[83,84,85]
	Estrogen: estradiol-17β	Antioxidant	Protecting ischemic brain injury and dopaminergic neurons in the substantia nigra.	[86,87]
Neurotransmitters	Serotonin and selective serotonin reuptake inhibitors (SSRIs)	Antioxidant, antidepressant, neuromodulatory	Controlling of mood, anxiety, sleep, improving depression.	[88,89,90,91,92]
	Dopamine and agonists	Antioxidant	Supporting the survival of nerve cells, promoting the synthesis of growth factors and neurogenesis	[50,93,94,95,96,97]
	Gamma-aminobutyric acid (GABA) and agonists	Antidepressant	Strengthening immunity, reducing anxiety and stress, improving insomnia and cognitive symptoms of schizophrenia patients.	[98,99]
	Acetylcholine	Antidepressant, inhibits Aβ accumulation	Controlling skeletal muscle and the autonomic nervous system, improving attention, memory, and learning, and protecting AD pathogenesis.	[100,101,102,103]
	Adrenaline and noradrenaline	Antioxidant, anti-apoptotic, anti-inflammatory	Affecting memory, movement, and attention, regulating neuroplasticity, and controlling glial activation.	[89]
Aminoacids and its derivatives	Creatine	Antioxidant, anti-apoptotic, anti-inflammatory	Protecting against hypoxic–ischemic and neurogenerative, reducing the loss of dopaminergic neurons for PD, improve motor performance for ALS.	[104,105,106,107,108]
	*N*-acetyl cysteine	Antioxidant	Controlling mood swings such as depression and bipolar disorder.	[109,110,111]
	Activated protein C	Anti-inflammatory, anti-apoptotic	Inhibiting the tumor suppressor protein p53 and preventing apoptosis in the hypoxic human brain	[112,113,114,115]
Proteins	Brain-derived neurotrophic factor	Anti-inflammatory, anti-apoptotic, antidepressant	Providing survival and plasticity of nerve cells, controlling depression and antidepressant mechanisms.	[116,117,118,119,120,121,122]
	Glial cell-derived neurotrophic factor	Anti-apoptotic	Reducing glutamate excitotoxicity, controlling depression and antidepressant mechanisms, and regulating neuroplasticity.	[115,123,124,125,126,127]
Others	Omega-3 fatty acids	Anti-inflammatory, antioxidant	Controlling cognitive development and sleep cycles, mood changes, improving neuropsychiatric disorders such as depression and schizophrenia, and regulating neuroplasticity.	[128,129,130]
	*N*-glycans	Anti-apoptotic, anti-inflammatory,	Supporting neurite migration, neurite growth, and synapse formation	[10,131,132,133,134]
	Cannabidiol	Anticonvulsant, antiepileptic, antidepressant, anti-inflammatory, antioxidant, anti-apoptotic	Reducing refractory seizure, anxiety, protein aggregation, improving motor and sensory impairment caused by ischemia, alleviating opioid withdrawal	[135,136,137,138]
